# Special Issue “Melanoma: From Molecular Pathology to Therapeutic Approaches”

**DOI:** 10.3390/ijms262211199

**Published:** 2025-11-19

**Authors:** Michael R. Eccles

**Affiliations:** 1 Department of Pathology, Dunedin School of Medicine, University of Otago, Dunedin 9016, New Zealand; michael.eccles@otago.ac.nz; 2 Maurice Wilkins Centre for Molecular Biodiscovery, Level 2, 3A Symonds Street, Auckland 1010, New Zealand

This Special Issue focuses on recent advances and innovations that have the potential to improve our understanding of the molecular pathology and therapeutic approaches associated with treating melanoma.

Globally, melanoma has a high incidence, accounting for the vast majority of skin cancer-related deaths [1]. Other forms of melanoma, such as uveal melanoma, also occur albeit more rarely, but they still contribute significantly to melanoma mortality [2]. Advanced melanoma is an aggressive cancer which is difficult to treat. Over the past two decades, there have been major advances in melanoma treatment, which include melanoma-targeted therapy as well as immune checkpoint inhibitor (ICI) immunotherapy, which have revolutionized melanoma treatment [3]. These advances have been driven by technical innovations and improved knowledge of molecular pathological biomarkers.

Despite recent therapeutic advances, a significant number of melanoma patients still do not benefit from these treatments [4,5]. In this issue, we have collated eight contributions, which can be accessed here: https://www.mdpi.com/journal/ijms/special_issues/XFL2P8303E (URL accessed on 7 November 2025). Their contributions to our knowledge of melanoma molecular pathology and therapeutic approaches are summarized in Figure 1. These papers address novel therapeutic approaches, as well as associated molecular pathologies, and they provide new angles for improving treatment outcomes for melanoma patients.

In the first contribution, Zanrè et al. investigated two antiretroviral drugs, doravirine and cabotegravir, and show that these drugs can influence apoptosis and cell proliferation in RAF-inhibitor-resistant melanoma cells, offering potential therapeutic strategies for overcoming drug resistance [Contribution 1].

Tura et al. focused on quercetin and showed that it impairs proliferation, viability, and metabolic activities while increasing oxidative stress in melanoma. The authors evaluated the effects of quercetin on the growth, survival, and glucose metabolism of the uveal melanoma cell line 92.1 [Contribution 2].

In the third contribution, Kuo et al. analyzed the transfer of mitochondria to melanoma cells from endothelial cells, which were found to increase M2-type macrophage polarization in a xenograft animal model, particularly through Nrf2/HO-1-mediated pathways. They found that the introduction of exogenous mitochondria from endothelial cells into melanoma cells promoted tumor growth [Contribution 3].

The fourth contribution by Liu et al. investigates Kinase Suppressor of RAS 1 (KSR1), which is a scaffolding protein for the RAS-RAF-MEK-ERK pathway. In this contribution, the authors examined the role of KSR1 in a BRAFV600E-transformed melanoma cell line, where they used CRISPR/Cas9 to knock out *KSR1*. Their results suggest that KSR1 directs ERK to phosphorylate substrates, which have a critical role in ensuring cell survival, and their results indicate that *KSR1* loss induces the activation of p38 Mitogen-Activated Protein Kinase (MAPK) and subsequent cell cycle aberrations and senescence [Contribution 4].

The fifth contribution by Hossain et al. is a review article that asks what research should focus on to improve clinical outcomes in immune checkpoint inhibitor therapy for metastatic melanoma. The review summarizes current immune checkpoint inhibitors for melanoma and the factors involved in resistance to treatment, and also discusses emerging evidence that the host microbiome can impact ICI treatment outcomes by modulating tumor biology and anti-tumor immune function [Contribution 5].

In the sixth contribution, Hossain et al. review genomic and epigenomic biomarkers of immune checkpoint immunotherapy response in melanoma. In this contribution, the authors review established and emerging biomarkers of ICI response, and they then provide a focus on epigenomic and genomic alterations with the potential to guide single-agent ICI immunotherapy or ICI immunotherapy in combination with other ICI immunotherapies or agents [Contribution 6].

In the seventh contribution Kerkour et al. carry out a systematic review and meta-analysis of publications in the literature which have reported genetic alterations in the primary cutaneous melanoma and the matched metastasis, and determined the extent of genetic concordance between them [Contribution 7].

Finally, the eighth contribution by Isaak et al. in this Special Issue brings together new ideas to develop personalized strategies for precisely battling malignant melanoma. The authors review the current landscape, explore personalized oncology techniques, and provide an up-to-date summary of the tools available to circumvent common barriers faced when battling melanoma [Contribution 8].

We sincerely thank all the authors who submitted their work and contributed to this collection, as well as the staff of the *International Journal of Molecular Sciences* for their invaluable support in making this editorial project a success. All articles are freely available through open access and distributed under the terms of the Creative Commons Attribution (CC BY) license 4.0 (https://creativecommons.org/licenses/by/4.0/, accessed on 19 November 2025).

## Figures and Tables

**Figure 1:**
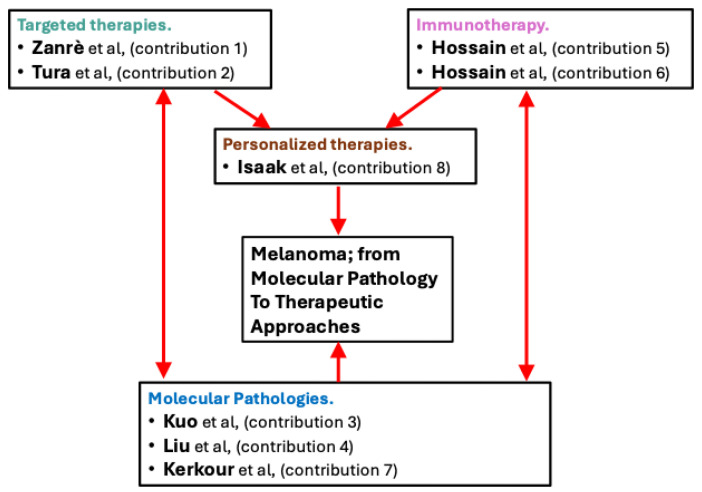
This figure summarizes the topics and papers in this Special Issue. The topics cover molecular pathologies, targeted therapies, and immunotherapy, which each have impacts on personalized therapies for melanoma. We have collected eight papers (Contribution 1 to Contribution 8) focused on different aspects of these topics, and which overall contribute to a better understanding of melanoma, from molecular pathology to therapeutic approaches.

## References

[1] Arnold M., Singh D., Laversanne M., Vignat J., Vaccarella S., Meheus F., Cust A.E., de Vries E., Whiteman D.C., Bray F. (2022). Global Burden of Cutaneous Melanoma in 2020 and Projections to 2040. JAMA Dermatol..

[2] Kaštelan S., Pavičić A.D., Pašalić D., Nikuševa-Martić T., Čanović S., Kovačević P., Konjevoda S. (2024). Biological characteristics and clinical management of uveal and conjunctival melanoma. Oncol Res..

[3] Qin Z., Zheng M. (2023). Advances in targeted therapy and immunotherapy for melanoma. Exp. Ther. Med..

[4] Holder A.M., Dedeilia A., Sierra-Davidson K., Cohen S., Liu D., Parikh A., Boland G.M. (2024). Defining clinically useful biomarkers of immune checkpoint inhibitors in solid tumours. Nat. Rev. Cancer.

[5] Mourah S., Louveau B., Dumaz N. (2020). Mechanisms of resistance and predictive biomarkers of response to targeted therapies and immunotherapies in metastatic melanoma. Curr. Opin. Oncol..

